# Clonality of CD4^+^ Blood T Cells Predicts Longer Survival With CTLA4 or PD-1 Checkpoint Inhibition in Advanced Melanoma

**DOI:** 10.3389/fimmu.2019.01336

**Published:** 2019-06-18

**Authors:** Akiko Arakawa, Sigrid Vollmer, Julia Tietze, Adrian Galinski, Markus V. Heppt, Maja Bürdek, Carola Berking, Jörg C. Prinz

**Affiliations:** Department of Dermatology and Allergology, University Hospital Munich, Ludwig-Maximilian-University Munich, Munich, Germany

**Keywords:** anti-tumor immunity, checkpoint inhibitor, CTLA4, melanoma, T cell clone, TCR, PD-1, T cell repertoire

## Abstract

Recognition of cancer antigens drives the clonal expansion of cancer-reactive T cells, which is thought to contribute to restricted T-cell receptor (TCR) repertoires in tumor-infiltrating lymphocytes (TILs). To understand how tumors escape anti-tumor immunity, we investigated tumor-associated T-cell repertoires of patients with advanced melanoma and after blockade of the cytotoxic T-lymphocyte-associated protein 4 (CTLA4) or programmed cell death 1 (PD-1). TCR Vβ-gene spectratyping allowed us to quantify restrictions of T-cell repertoires and, further, diversities of T-cell clones. In this study, we show that the blood TCR repertoires were variably restricted in CD4^+^ and extensively restricted in CD8^+^ T cells of patients with advanced melanoma, and contained clones in both T-cell fractions prior to the start of immunotherapy. A greater diversification especially of CD4^+^ blood T-cell clones before immunotherapy showed statistically significant correlations with long-term survival upon CTLA4 or PD-1 inhibition. Analysis of TILs and corresponding blood available in one patient indicated that blood clonality may at least partially be related to the clonal expansion in the tumor microenvironment. In patients who developed severe immune-related adverse events (IrAEs), CD4^+^ and CD8^+^ TCR spectratypes became more restricted during anti-CTLA4 treatment, suggesting that newly expanded oligoclonal T-cell responses may contribute to IrAEs. This study reveals diverse T-cell clones in the blood of melanoma patients prior to immunotherapy, which may reflect the extent to which T cells are able to react against melanoma and potentially control melanoma progression. Therefore, the T-cell clonality in the circulation may have predictive value for antitumor responses from checkpoint inhibition.

## Introduction

Anti-tumor T-cell responses can control clinical progression of cancer, as exemplified by the effects of T-cell targeting immunotherapies such as immune checkpoint inhibition, including antibodies against cytotoxic T-lymphocyte-associated protein 4 (CTLA4) or programmed cell death 1 (PD-1), high-dose IL-2 treatment, or adaptive T-cell immunotherapy (1–3). In the natural course of tumor progression without immunotherapy, however, T cells usually fail to impede tumor progression. Anti-CTLA4 and anti-PD-1 therapies can prolong survival rates of patients with advanced melanoma, but also induce organ-specific toxicities in a substantial number of patients, termed immune-related adverse events (IrAEs), which restrict the long-term benefits from this immunotherapeutic approach (1, 2). CTLA4, which is expressed mainly on CD4^+^ T cells after TCR-mediated activation, suppresses TCR-driven activation and proliferation through interfering with costimulatory CD28 signaling by antigen presenting cells (4). Thus, inhibition of CTLA4 is thought to lower the threshold for anti-tumor T-cell activation/proliferation *via* increasing CD28 signaling (4). PD-1 is a cell surface receptor that inhibits effector functions of antigen-specific T cells upon ligand binding (5, 6). Since PD-1 inhibition directly modulates functions of various typed cells expressing PD-1 (6), CTLA-4 and PD-1 blockade are thought to exert distinctive immune mechanisms (7). It is not fully understood why T cells fail to inhibit tumor growth without immunotherapies and why a significant subgroup of patients does not respond to CTLA4 or PD-1 blockade.

Upon recognizing antigens, antigen-reactive T cells are activated and proliferate, a process leading to clonal expansion (8). Tumor recognition by T cells is impaired in cancer patients (9). Nevertheless, tumor-specific T cells occur responding to tumor antigens that include individual “neoantigens” derived from mutated proteins in cancer cells (10–13). These tumor-specific T cells however, may remain anergic (10). T-cell clones can be tracked by determining T-cell receptor (TCR) rearrangements composed of variable (V)-diversity (D)-joining (J) region genes, which generate the antigen-specific complementarity determining region 3 (CDR3). Analysis of T-cell clonality may therefore reveal the degree of tumor-antigen driven T-cell expansions and help to dissect mechanisms underlying T-cell tolerance to cancer antigens.

Interpretation of complexity of T-cell repertoires in view of antigen specificities with a potential diversity of −10^18^ different TCRs is still challenging, although various analyses technologies and measures have been developed (14). CDR3 spectratyping, by the immunoscope technology, can visualize T-cell repertoires for each V-gene family according to CDR3 size. The immunoscope technology revealed T-cell repertoire restrictions related with various immune conditions (14, 15), though it has not been widely applied to characterize TCR repertoires in melanoma patients. Spectratyping of total blood T cells from two patients with advanced malignant melanoma had shown only minor TCR repertoire restrictions (16), supporting a long-held assumption that tumor-induced T-cell repertoire restrictions are confined to the tumor microenvironment only, without affecting blood TCR diversity. As an alternative mode of TCR analysis, high throughput sequencing of TCRs generates large data sets of TCR usage (14). Indeed, several studies have provided important insights for T-cell dynamics in blood of melanoma patients under CTLA4 blockade (17–19). These studies employed several parameters for data interpretation such as “richness” (total number of unique clones), “eveness” that reflects how similar the frequencies of clones are to each other, or comparison of each clone numbers before and after CTLA4 inhibition. Cha et al. reported that lesser decreases in numbers of decreased T-cell clones in the blood were associated with favorable response to CTLA4 inhibition (17), suggesting the importance of pre-existing tumor specific T-cell clones for anti-tumor response under CTLA4 blockade. In contrast, Postow et al. reported that higher richness and evenness reflecting diverse TCR repertoires before treatment were associated with clinical benefit under CTLA4 blockade, which are considered to reflect a diverse TCR repertoire (19). Thus, these observations in the two major studies seemingly provide apparently contradicting views.

Several observations suggest that pre-existing T-cell responses are associated with better outcome of both anti-CTLA4 and anti-PD-1 therapy (20, 21). T-cell responses against certain tumor antigens were detected before initiation of CTLA4 blockade in patients responding well to the immunotherapy (20). Dense CD8^+^ T-cell infiltration in the tumor microenvironment correlated with better prognosis under PD-1 inhibition (21). Increased mutational load in tumors has been correlated with better responsiveness to CTLA4 or PD-1 blockade, suggesting that T-cell responses against cancer neoantigens are enhanced (12, 13).

T-cell clone analyses are recently considered as useful for early diagnoses of IrAEs. It has been shown using next generation sequencing that patients who experienced severe IrAE under CTLA4 inhibitor exhibited higher numbers of T-cell clones expanding after the treatment even before the clinical symptoms of IrAEs (18, 22, 23).

Despite these insights, it remained elusive whether blood T-cell repertoires in melanoma patients differ from healthy conditions and thus have potential prognostic values or not. To address this issue, we performed a detailed analysis of T-cell repertoires in patients with advanced melanoma using CDR3 size spectratyping and sequencing of TCR rearrangements in combination with CD4/CD8 T-cell separation (15). Taking advantage of visualized TCR repertoires, we applied novel measures to quantify TCR restrictions and clonalities by stratifying clonal T-cell populations according to CDR3 lengths and TCR-Vβ gene usage (15), both of which substantially contribute to antigen specificity (24). Moreover, we differentially analyzed CD4^+^ and CD8^+^ T cells, as the two main T-cell subsets differentially recognize antigens *via* HLA-class II or class I molecules, and exert definite effector functions in the immunological tumor control (25). Indeed, these measures had allowed us to identify disease specific pathomechanisms of HLA-class II related CD4^+^ T-cell mediated autoimmunity in a most severe subset of psoriasis, generalized pustular psoriasis (15). In the blood of melanoma patients, our spectratyping analyses now precisely reveal substantial repertoire restrictions with clonal T-cell expansions in both CD4^+^ and CD8^+^ T cell subsets. Notably, broader clonal expansions of blood T cells before treatment predicted longer survival times of melanoma patients following CTLA4 inhibition and were also observed in long-term survivors from anti PD-1 therapy. We conclude that repertoire analyses of blood T-cell clones may predict clinical responses to blockade of CTLA4 as well as of PD-1.

## Patients and Methods

### Patients

Twenty patients with pathologically confirmed diagnosis of unresectable stage IV melanoma and healthy individuals (*n* = 8) participated voluntarily and gave written informed consent (Table 1). Patients were more than 18 years of age and had normal hematologic and organ function and an Eastern Cooperative Oncology Group status of 0 or 1. Patients with preexisting systemic autoimmune diseases and active infection were excluded. Healthy donors were defined as individuals without a history of cancer or autoimmune diseases. Patients and healthy control groups had the same female/male ratio. The median age was 60.5 in patients, 37 in control group. The study was performed in accordance with the Declaration of Helsinki and approved by the Ethics Committee of the Ludwig-Maximilian-University, Munich.

**Table 1:** Characteristics of analyzed patients.

**Pt**.	**Age**	**Sex**	**Tumor stage**	**IT**	**Cycles of IT**	**Objective response (12 Wks)**	**Objective response (24 Wks)**	**Survival after IT (Months)**	**IrAE in IT**
1	69	F	M1c(0)	lpilimumab	4	PD	PR	14	None/mild
2	56	M	M1b(0)	lpilimumab	4	PD	SD	29	None/mild
3	54	M	M1c(0)	lpilimumab	4	PD	Death	3.25	Severe
4	57	M	M1c(0)	lpilimumab	4	SD	SD	58	None/mild
5	36	F	M1c(0)	lpilimumab	4	SD	PD	53	None/mild
6	59	M	M1c(0)	lpilimumab	4	SD	SD	58	None/mild
7	31	F	M1a(0)	lpilimumab	2	SD	PD	27	None/mild
8	69	F	M1c(1)	lpilimumab	2	PD	Death	3	None/mild
9	71	M	M1a(0)	lpilimumab	2	PD	Death	3.5	Severe
10	67	F	M1c(0)	lpilimumab	3	PD	PD	9	Severe
11	73	M	M1b(1)	lpilimumab	4	PD	PD	12	None/mild
12	39	F	M1a(0)	lpilimumab	4	PD	PD	15	Severe
13	62	F	M1a(0)	lpilimumab	4	PD	PD	36	None/mild
14	72	M	M1b(0)	lpilimumab	4	PR	SD	49	None/mild
15	69	M	M1c(0)	lpilimumab	4	PD	PD	5	None/mild
16	52	M	M1b(0)	lpilimumab	4	PD	PD	39	None/mild
17	53	F	M1c(1)	lpilimumab	3	PD	Death	3.17	None/mild
18	63	F	M1c(0)	Pembrolizumab	13	PR	PR	51	None/mild
19	74	F	M1b(0)	Pembrolizumab	32	PR	PR	54	None/mild
20	58	M	M1c(0)	Pembrolizumab	35	SD	SD	50	None/mild

*Tumor is staged according to AJCC (8th edition). IT, Immunotherapy; IrAE, Immune related adverse event, PR, Partial response; SD, Stable disease; PD, Progressive disease*.

CTLA4 blockade with ipilimumab was given to 17 patients at 3 mg/kg body weight every 3 weeks for a maximum of 4 cycles. The post-treatment blood samples were taken 3–5 weeks after final infusion of ipilimumab (*n* = 13). PD-1 inhibitor pembrolizumab was given to 3 patients within the trial MK-3475-006/KEYNOTE-006 at 10 mg/kg body weight every 2 (Q2W) or 3 (Q3W) weeks. Pembrolizumab treatment was continued until disease progression or unacceptable toxicity or was maintained for a maximum of 2 years if a radiologic response according to RECIST1.1 was achieved. Overall survival was defined as the elapsed time between the first ipilimumab/ pembrolizumab treatment and either the date of death or the last follow-up, if death was not observed during the follow-up period. All deaths were related to melanoma. Toxicity was assessed using Common Terminology Criteria for Adverse Events, version 3.0. Grade 3–4 events were assigned as “severe” IrAEs.

### TCR β-Chain Repertoire Analysis

Peripheral blood mononuclear cells (PBMC) were prepared from heparinized blood by density gradient centrifugation. CD4^+^ or CD8^+^ cells were purified from freshly isolated PBMC with > 95% purity using antibody-coated magnetic beads (Dynal Biotech). Tumor infiltrating lymphocytes (TILs) were separated by mechanical digestion (26). CDR3 fragment spectratyping, cloning and sequencing of Vβ-gene transcripts was performed as described (15). Gaussian patterns of CDR3 fragment length are defined by more than 5 peaks at 3 bp intervals in a bell-shaped size distribution. Peaks more than 1.5-fold higher than expected from a Gaussian pattern were defined as “distinct” peaks. Vβ-chain families that had distinct peaks or did not show Gaussian patterns were assigned as “restricted.” Spectratypes were printed out with a defined width and length range for each Vβ chain, and analyzed on the printouts, three times independently without knowledge of the clinical course or prior assessments, yielding similar results. Arden nomenclature was used for Vβ-specification (27).

### Statistical Analysis

As there was more than one group without normal distributions in each comparison (Shapiro-Wilk *W*-Test), two-group comparison was performed using Mann-Whitney *U*-test. The Pearson rank correlation test was used to calculate the correlation coefficient between CD4^+^ and CD8^+^ T cells. Two-tailed *p* < 0.05 was considered significant. The probability of overall survival was estimated by the Kaplan-Meier method. All statistical analyses were performed using GraphPad version 4 and confirmed by R software. Sample size was determined based on observed effect sizes. No samples were excluded from analysis.

## Results

### Restricted TCR Repertoires of CD4^+^ and CD8^+^ Blood T Cells in Melanoma Patients

**Figure 1:**
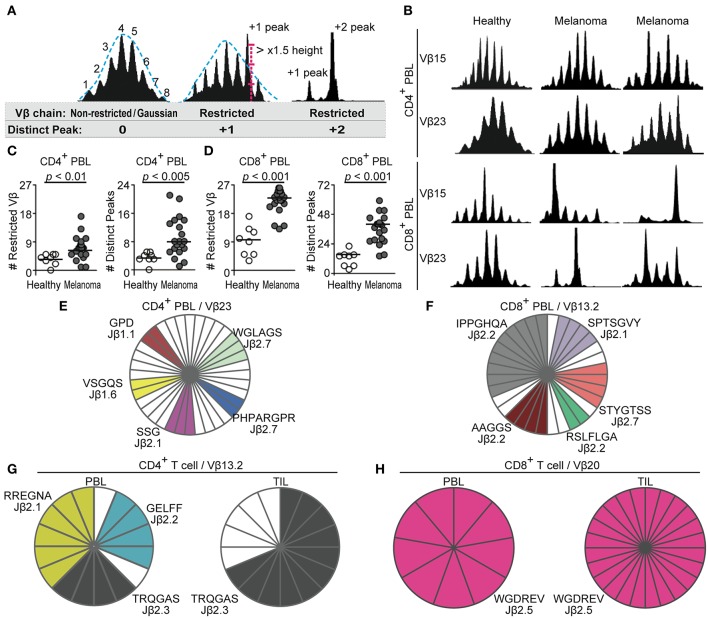
CD4^+^ and CD8^+^ blood T cells of melanoma patients show TCR repertoire restrictions with clonal expansions. **(A)** Definitions of non-restricted/restricted Vβ-chain and distinct peak. **(B)** Representative CDR3 spectratypes determined for 27 different Vβ-gene families from CD4^+^ and CD8^+^ peripheral blood lymphocytes (PBL) of healthy controls (*n* = 8) and patients with advanced melanoma (*n* = 20). Vβ15 spectratyping is shown in 27 bp width and Vβ23 in 24 bp width. **(C,D)** Numbers of restricted Vβ-gene families or distinct peaks in spectratypes of CD4^+^ **(C)** and CD8^+^ **(D)** blood T cells are compared by Mann-Whitney *U*-test. Each dot represents one subject. **(E,F)** Sequencing of TCR β-chain rearrangements from select TCR Vβ-gene families identified clonal TCRs in CD4^+^ **(E)** and CD8^+^ **(F)** blood T cells of melanoma patients. Each slice represents a TCR Vβ-gene rearrangement. White slices represent sequences found only once. Identical TCR rearrangements are color-highlighted. CDR3 amino acid sequences (one letter code) and rearranged Jβ genes are given. **(G,H)** Sequencing of TCR β-chain rearrangements of CD4^+^ **(G)** and CD8^+^ **(H)** cells from blood and tumor-infiltrating lymphocytes (TILs) identified common clones.

We first examined TCR repertoires in 20 patients with metastatic melanoma before the start of immunotherapy as compared to 8 healthy individuals. Whole TCR repertoires of CD4^+^ or CD8^+^ blood T cells were analyzed by CDR3 size spectratyping of 27 Vβ-chain families. TCR Vβ-gene families that had distinct peaks over assumed Gaussian height or did not assume Gaussian patterns were defined as “restricted” (Figure 1A). Before immunotherapy, TCR repertoires of both CD4^+^ and CD8^+^ T cells in melanoma patients were significantly more restricted compared to those from healthy controls (Figures 1A–D). While in healthy individuals the majority of TCR Vβ-gene families of CD4^+^ T cells showed Gaussian distributions typical of unselected T-cell populations, significantly higher numbers of TCR Vβ-gene families of CD4^+^ blood T cells in melanoma patients exhibited repertoire restrictions (Figures 1B,C, *p* = 0.0056). The spectratypes of CD4^+^ T cells from melanoma patients showed significantly greater numbers of distinct peaks than healthy controls (Figures 1B,C, *p* = 0.0018), indicating overrepresentation of TCR rearrangements with a particular length. CD8^+^ blood T cells from melanoma patients also displayed markedly higher numbers of restricted TCR Vβ-gene families (*p* = 0.0000028) and distinct peaks than healthy individuals (Figures 1B,D, *p* = 0.000010). Thus, the comparison with healthy controls documents to which extend clonal expansions actually occur in progressive melanoma and clarify the relationship with tumor development.

To confirm that restricted spectratypes corresponded to T-cell clones, i.e., repetitive T cells with identical TCRs derived from a common progenitor T cell, we cloned and sequenced TCR β-chain rearrangements of select TCR Vβ-gene families. Various clonal TCRs in CD8^+^ and CD4^+^ blood T cells from melanoma patients were identified (Figures 1E,F), while the same approach did not detect redundant TCRs in the TCR Vβ-gene families of healthy individuals (data not shown).

### Identical T-Cell Clones in TILs and Blood Lymphocytes in Melanoma

TILs are considered to directly exert anti-tumor responses (28, 29). We examined T-cell clonalities of selected Vβ-gene families in blood lymphocytes and TILs of one patient by sequencing. This analysis identified several identical TCR rearrangements that were clonally expanded in T cells from both blood and TILs. Three clones with Vβ13.2 TCR rearrangements were expanded in CD4^+^ circulating T cells, and one of the clones was highly expanded in CD4^+^ TILs (Figure 1G). When Vβ-gene families of CD8^+^ TILs were dominated by a clonal TCR, the same TCR rearrangement was dominantly identified in CD8^+^ blood lymphocytes of the same patient (Figure 1H). Thus, CD4^+^ and CD8^+^ blood T-cell clones were enriched in the corresponding tumor microenvironment of the same patient. Identical CD8^+^ clones in TILs and the blood have been recently observed (30). Our data now expand the insights into tumor-associated T-cell diversity by showing that not only CD8^+^ but also CD4^+^ T cells which had experienced antigen-driven clonal expansion are distributed through blood circulation of melanoma patients.

### Restricted TCR Repertoires in Melanoma Patients Were not Different According to Age, Sex, Tumor Stage, and Pretreatment by Dacarbazine

**Figure 2:**
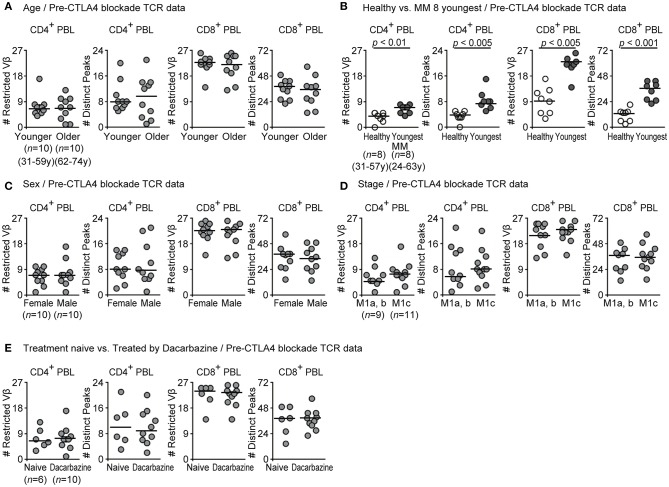
Pretreatment TCR repertoire restrictions were not different in patient groups with different ages, sex, metastatic organ, or dacarbazine treatment. **(A,C,D,E)** Numbers of restricted Vβ-gene families or distinct peaks in spectratypes of CD4^+^ and CD8^+^ blood T cells are compared between patient groups with different age **(A)**, sex **(C)**, stage **(D)** or preimmunotherapy dacarbazine treatment **(E)** by Mann-Whitney *U*-test. Data in Figures 1C,D are re-analyzed. **(B)** TCR repertoires are compared between healthy controls and 8 youngest melanoma patients so that there are no statistical differences in age between groups.

Our cohort may have potential confounding factors to affect TCR repertoires (14, 31). The patients were older than the normal donors. Still, when we divided patients into two groups by the mean age, the TCR data did not show statistically significant differences between younger and older melanoma patients in our analyses (Figure 2A, CD4, Restricted Vβ-gene families, *p* = 0.47; CD4, Distinct peaks, *p* = 0.55; CD8, Restricted Vβ-gene families, *p* = 0.52; CD8, Distinct peaks, *p* = 0.62). When we selected the 8 youngest patients (*n* = 8 age 31–57), there were no statistical significant differences in age between this group and healthy controls. Yet, the differences between TCR data of healthy controls and the youngest melanoma group remained statistically significant (Figure 2B, CD4, Restricted Vβ-gene families, *p* = 0.0073; CD4, Distinct peaks, *p* = 0.0010; CD8, Restricted Vβ-gene families, *p* = 0.0011; CD8, Distinct peaks, *p* = 0.00075). Patients groups divided by sex, stage, and pretreatment with dacarbazine showed similar TCR restrictions (Figures 2C–E). These findings indicate that age, sex, affected organs, and pretreatment by dacarbazine did not have major effects on the observed repertoire restrictions in these melanoma patients.

### CD8^+^ Blood T-Cell Repertoire Were More Restricted Than CD4^+^ Blood T Cells in Melanoma Patients

We compared the level of repertoire restrictions between CD4^+^ and CD8^+^ blood T cells within each individual. Consistent with previous reports (31), CD8^+^ T-cell repertoires in healthy individuals showed more extensive restrictions than corresponding CD4^+^ T cells, in terms of numbers of restricted TCR Vβ-gene families and distinct peaks (Supplementary Figure 1A, *p* = 0.025, *p* = 0.0092, respectively). As in healthy controls, CD8^+^ T-cell repertoires of melanoma patients were more restricted than those of CD4^+^ T cells (Supplementary Figure 1B, *p* = 0.000063, *p* = 0.000064, respectively).

We then investigated if antigen-specific CD4^+^ and CD8^+^ T-cell responses were correlated. In healthy individuals, numbers of restricted TCR Vβ-gene families or distinct peaks of CD4^+^ and CD8^+^ T cells did not correlate with each other (Supplementary Figure 1C, *p* = 0.93, *p* = 0.62, respectively). In contrast, in melanoma patients, numbers of restricted TCR Vβ-gene families showed statistically significant positive correlations between CD4^+^ and CD8^+^ T cells (Supplementary Figure 1D, *p* = 0.0026). Distinct peak numbers in CD4^+^ and CD8^+^ T cells tended to correlate as well (Supplementary Figure 1D, *p* = 0.078). These findings indicated an overall increased antigen-induced selection pressure in both T-cell subsets of melanoma patients.

### Effects of CTLA4 Blockade on TCR Diversity in Melanoma Patients

**Figure 3:**
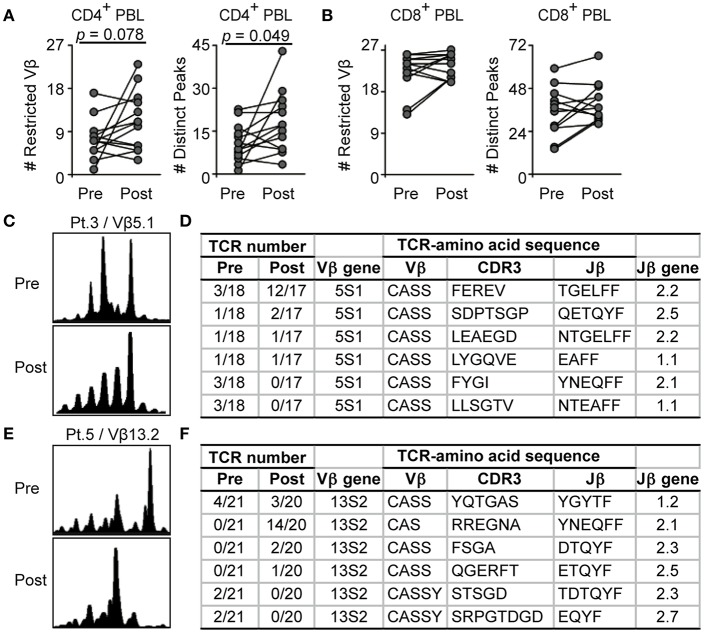
CD4^+^ T-cell repertoire restrictions of melanoma patients are more affected by CTLA4 blockade than CD8^+^ T cells. **(A,B)** Restricted Vβ-gene families or distinct peaks in CD4^+^ **(A)** and CD8^+^ **(B)** blood T cells were compared before and after CTLA4 blockade by Wilcoxon rank sum test. Values of each individual are connected by lines (*n* = 13). Pretreatment data are also shown in Figures 1C,D. **(C–F)** Spectratyping of Vβ5.1- **(C)** and Vβ13.2-gene families **(E)** in CD8^+^ blood T cells of melanoma patients and corresponding sequencing of TCRβ-chain rearrangements before and after CTLA4 blockade **(D,F)**. CDR3 amino acid sequences and rearranged Jβ genes are given. TCR number indicates identical among total sequences **(D,F)**.

CTLA4 blockade is known to alter TCR repertoires in whole blood T cells (17, 18). We then analyzed how CTLA4 blockade might affect TCR repertoires in the CD4^+^ and CD8^+^ T-cell subsets in 17 melanoma patients treated with CTLA4 antibody. Following CTLA4 blockade, numbers of restricted TCR Vβ-gene families and distinct peaks tended to increase in CD4^+^ blood T cells as compared to pretreatment values (*p* = 0.078, *p* = 0.049, respectively), but not in CD8^+^ blood T cells (Figures 3A,B, *p* = 0.11, *p* = 0.17, respectively). These findings indicated that CTLA4 blockade particularly broadened antigen-driven CD4^+^ T-cell responses. This is consistent with the previous observation that CTLA4 is primarily acting on antigen-specific responses of CD4^+^ T cells, although this effect had not been examined regarding T-cell clonality (4, 32).

We further analyzed the frequencies of distinct CD8^+^ T-cell clones before and after CTLA4 blockade. In CD8^+^ T cells of patient 3, Vβ5.1 spectratyping revealed a distinct peak before treatment which was maintained after CTLA4 blockade (Figure 3C). Vβ5.1 T-cell clones showed different kinetics: one clone was significantly increased in number (Figure 3D, FEREV), 3 clones were maintained (Figure 3D, SDPTSGP, LEAEGD, LYGQVE), and 2 clones were not detected any more after the treatment (Figure 3D, FYGI, LLSGTV). In patient 5, the Vβ13.2 spectratype of CD8^+^ T cells was restricted, with one distinct peak before immunotherapy which was lost after CTLA4 blockade (Figure 3E). In Vβ13.2, one persistent clone was present before and after treatment (YQTGAS). Three clones emerged (Figure 3F, RREGNA, FSGA, QGERFT), while two other clonal rearrangements apparently disappeared (Figure 3F, STSGD, SRPGTDGD). Despite the limited numbers of sequences, these analyses revealed that certain clones were maintained in the blood during CTLA4 inhibition, as reported (17).

### Degrees of Repertoire Restrictions in CD4^+^ and CD8^+^ Blood T Cells Were Correlated in Melanoma Patients

Given that CTLA4 blockade had different impacts on repertoire restrictions of CD4^+^ and CD8^+^ T cells (Figure 3), we analyzed the correlation between CD4^+^ and CD8^+^ T-cell repertoire restrictions after anti-CTLA4 treatment. While correlation of restricted TCR Vβ-gene families somewhat decreased after immunotherapy (Supplementary Figure 1E, *p* = 0.062), correlation of the numbers of distinct peaks between CD4^+^ and CD8^+^ T cells became statistically significant (Supplementary Figure 1E, *p* = 0.0030). Together, degrees of repertoire restrictions in CD4^+^ and CD8^+^ blood T cells showed positive correlations with each other in melanoma patients both before and after CTLA4 blockade (Supplementary Figures 1D,E).

### Diversified CD4^+^ Blood T-Cell Clones Prior to Immunotherapy May Predict Clinical Response to CTLA4 Blockade

**Figure 4:**
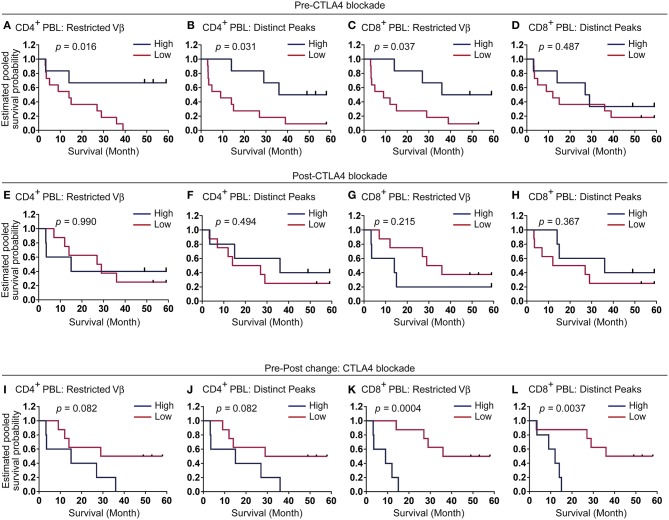
Melanoma patients with more restricted CD4^+^ blood T-cell repertoires gain longer survival under CTLA4 blockade. **(A–L)** Kaplan-Meier survival curves according to the numbers of restricted Vβ-gene families or distinct peaks in CD4^+^ **(A,B,E,F,I,J)** and CD8^+^ **(C,D,G,H,K,L)** blood T cells before, after CTLA4 blockade, or changes pre-post CTLA4 blockade. Blue lines represent top tertile (pre-CTLA4 blockade *n* = 6, post-CTLA4 blockade *n* = 5, pre-post change CTLA4 blockade *n* = 5), and the rest patients were depicted by red (pre-CTLA4 blockade *n* = 11, post-CTLA4 blockade *n* = 8, pre-post change CTLA4 blockade *n* = 8). The threshold value was 7 **(A)**, 9.3 **(B)**, 23.7 **(C)**, 40 **(D)**, 13 **(E)**, 20 **(F)**, 26 **(G)**, 43 **(H)**, 4 **(I)**, 8 **(J)**, 4 **(K)**, and 7 **(L)** for each panel.

We further examined if TCR repertoire restrictions correlate with clinical responses to CTLA4 blockade. Indeed, patients with greater numbers of restricted TCR Vβ-gene families in their CD4^+^ blood T cells before immunotherapy survived significantly longer than patients with lesser restricted Vβ-families (Figure 4A, *p* = 0.016). Patients who exhibited more distinct peaks in CD4^+^ blood T-cell repertoires before the treatment showed longer survival times than patients with lower numbers (Figure 4B, *p* = 0.031). Higher numbers of restricted TCR Vβ-gene families in CD8^+^ blood T cells before treatment were associated with longer overall survival (Figure 4C, *p* = 0.037), while numbers of distinct peaks in CD8^+^ blood T cells showed no correlation with overall survival (Figure 4D, *p* = 0.49). Overall survival rates were not different when assessed regarding TCR repertoires after CTLA4 blockade (Figures 4E–H), while T-cell repertoires tended to become more restricted in CD4^+^ blood T cells after CTLA4 treatment (Figure 3A). Moreover, the pretreatment numbers of restricted TCR Vβ-gene families and distinct peaks tended to correlate with objective response at 12 weeks (Supplementary Figure 2A). The repertoires in CD4^+^ and CD8^+^ blood T cells before CTLA4 blockade were significantly more restricted in patient with better objective responses at 24 weeks as compared to patients with poorer responses (Supplementary Figure 2B), further supporting the prognostic values of TCR repertoire analyses.

Changes of CD4^+^ TCR repertoires from pre- to post-immunotherapy were statistically not correlated with overall survivals (Figures 4I,J, restricted TCR Vβ-gene families *p* = 0.082, distinct peaks *p* = 0.082). Instead, increasing numbers of restricted TCR Vβ-gene families and distinct peaks of CD8^+^ T cells were rather correlated with significantly decreased survivals (Figures 4K,L, *p* = 0.00042, *p* = 0.0037, respectively).

These results demonstrated that degrees of pretreatment TCR repertoire restrictions, especially in CD4^+^ blood T cells may have prognostic values for clinical responses to CTLA4 inhibition.

The pretreatment CD8^+^ T-cell repertoire restrictions were lesser predictive for overall survival following CTLA4 inhibition than that of CD4^+^ T cells (Figures 4A–D), though independent effects may have to be verified in larger cohorts, given the correlation of restricted TCR Vβ-gene families between CD4^+^ and CD8^+^ T cells (Supplementary Figure 1D). In contrast, CD8^+^ T-cell repertoires which become more restricted in the blood during CTLA4 inhibition may indicate poorer prognosis.

### T-Cell Repertoires Became More Restricted With Severe IrAEs Under CTLA4 Blockade

Four patients experienced severe IrAEs in our cohort. There were no statistical differences between patients without/with severe IrAEs in numbers of restricted TCR Vβ-gene families and distinct peaks in CD4^+^ T cells before treatment (Supplementary Figure 3A, *p* = 0.19, *p* = 0.11, respectively). CD8^+^ T cells of patients who developed IrAEs showed a tendency for fewer restricted TCR Vβ-gene families and distinct peaks before treatment (Supplementary Figure 3A, *p* = 0.098, *p* = 0.079, respectively). Posttreatment values of restricted TCR Vβ-gene families and distinct peaks in both CD4^+^ or CD8^+^ T cells did not differ according to severe IrAEs (Supplementary Figure 3B).

**Figure 5:**
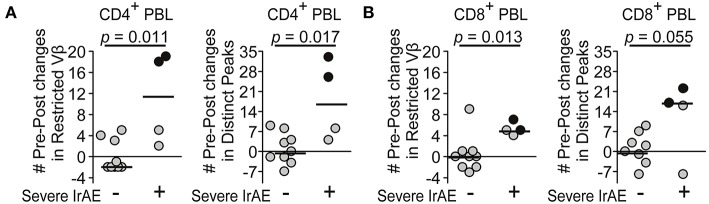
T-cell repertoires became more restricted during CTLA4 blockade in patients who experienced severe immune-related adverse events (IrAEs). **(A,B)** Changes in numbers of restricted Vβ-gene families or distinct peaks in CD4^+^ (**A**) and CD8^+^ **(B)** blood T cells during CTLA4 blockade were compared between patients with/without severe IrAE by Mann-Whitney *U*-test (*n* = 9, 4, respectively). Black dot represents patient with severe colitis.

The numbers of restricted TCR Vβ-gene families and distinct peaks in CD4^+^ (*p* = 0.011, *p* = 0.017, respectively) as well as CD8^+^ T cells (*p* = 0.013, *p* = 0.055, respectively) had increased more in anti-CTLA4 treated patients with severe IrAEs than in patients without severe IrAEs (Figure 5). These results are consistent with previous studies showing that newly expanded T-cell clones correlated with severe IrAEs from CTLA4-blockade (18, 22, 23) This study included two patients who experienced severe colitis under CTLA4 blockade (black dot, Figure 5) and had the most prominent increases in CD4^+^ T-cell repertoire restrictions (black dot, Figure 5).

### Pre-existing Clonal CD4^+^ T-Cell Responses Correlated With Longer Survival Times With PD-1 Blockade

**Figure 6:**
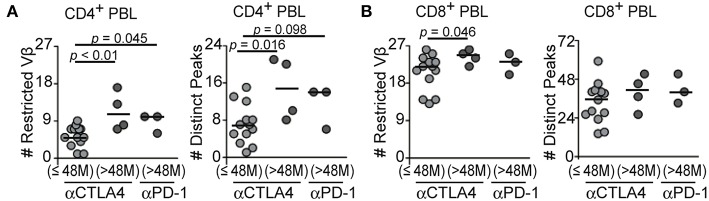
Pretreatment T-cell repertoire restrictions in patients who survived shorter or longer than 48 months under CTLA4 and PD-1 blockade. **(A,B)** Numbers of restricted Vβ-gene families or distinct peaks in CD4^+^ **(A)** and CD8^+^ **(B)** blood T cells before CTLA4 blockade were analyzed according to survival duration of less (CTLA4 blockade, *n* = 13) and more than 48 months (CTLA4 or PD-1 blockade, *n* = 4, 3 respectively). When *p*-value of Kruskal-Wallis *H*-test was significant, two group comparison was performed by Mann-Whitney *U*-test.

Blockade of PD-1 can increase survival rates of patients with advanced melanoma (2). Three patients in our cohort were treated with anti-PD-1 antibody. All of them survived longer than 48 months under anti-PD-1 treatment. To assess the prognostic relevance of pre-existing T-cell clones, we first divided the anti-CTLA4-treated patients into two groups by their survival times. Patients who survived longer than 48 months with CTLA4 inhibition showed significantly higher numbers of restricted TCR Vβ families, distinct peaks in CD4^+^ T blood T cells (*p* = 0.0084, *p* = 0.016, respectively), and restricted TCR Vβ-gene families in CD8^+^ T blood T cells before treatment (*p* = 0.046), compared to shorter survivors with CTLA4 blockade (Figures 6A,B), further supporting the prognostic values of pretreatment TCR repertoires, in accord with the results from Kaplan-Meyer analyses (Figure 4).

In the long-term survivors under anti-PD-1 treatment, numbers of restricted TCR Vβ-gene families in CD4^+^ T blood T cells were significantly higher than in short-term survivors with CTLA4 inhibition (Figure 6A, *p* = 0.045). Patients who survived longer than 48 months with PD-1 blockade tended to have greater numbers of distinct peaks in CD4^+^ T blood T cells than short-term survivors with anti-CTLA4 treatment, though the difference did not reach statistical significance (Figure 6A, *p* = 0.098). The numbers of restricted TCR Vβ-gene families and distinct peaks in CD8^+^ blood T cells before treatment were not different in long-term survivors with anti-PD-1 treatment (Figure 6B, *p* = 0.28, *p* = 0.18, respectively). These findings suggested that broader clonal CD4^+^ T-cell responses may also favor longer survivals under PD-1 inhibition.

## Discussion

CDR3 spectratyping can visualize T-cell repertoires by stratifying the clonal T-cell responses according to CDR3 size for each Vβ-gene family. Combined with CD4/CD8 separation, our analyses now revealed that CD4^+^ and CD8^+^ T-cell repertoires in circulation of patients with advanced melanoma were restricted, with T-cell clones that were diversified in terms of Vβ-gene family and CDR3 length. In contrast to highly restricted repertoires of CD8^+^ T cells, which were seen in all analyzed patients, we observed that the degree of CD4^+^ blood T-cell repertoire restrictions varied among melanoma patients. Notably, several CD4^+^ or CD8^+^ clones in the blood were also present in the tumor microenvironment. This result suggests that T cell clones circulating in the blood may correspond to TILs, some of which may have expanded in response to tumor antigens (10–13, 20). The TCR repertoire restrictions in blood of melanoma patients in this study likely correspond to those circulating clonal T-cell populations previously detected by next generation sequencing (17–19). Consistent with these findings, we have previously reported increased frequencies of memory/antigen experienced-phenotyped CD45RO^+^ CD4^+^ T cells in the blood of melanoma patients (26). These findings may advance our basic understanding of how diverse the circulating tumor-associated T-cell clones are, and how metastatic melanoma potentially alters systemic T-cell activation and differentiation.

As a particular finding, the data obtained this way demonstrate that broader T-cell responses with a greater diversity of CD4^+^ blood T-cell clones may predict longer survival of melanoma patients treated with anti-CTLA4 or PD-1 antibodies. This insight is made possible by analyses of T-cell clonality in view of restricted TCR Vβ-gene families and CDR3 size. Although our study is potentially limited by the small sample size, our data are supported by previous observations showing that pre-existing tumor-specific T cells are relevant for the anti-tumor responses after CTLA4 or PD-1 inhibition (17, 20, 21). Several studies showed that increased frequencies of memory T cells or decreased frequencies of naïve-phenotype CD4^+^ or CD8^+^ T cells in the blood were correlated with better outcomes in anti-CTLA4-treated melanoma patients (33–35). Our results emphasize a critical role of clonally expanded and antigen-experienced CD4^+^ T cells in tumor control. This may be consistent with the mechanism of action of CTLA4 inhibition, which primarily acts on CD4^+^ T cells (4, 32). The clonal CD4^+^ T-cell expansions likely manifest higher frequencies of central memory CD4^+^ T cells which had been observed in blood of two melanoma patients who responded well to PD-1 blockade (36). Further analysis in greater numbers of anti-PD-1 treated patients will be required to clarify this point.

Our findings from a different mode of TCR repertoire analyses and stepwise approach may reconcile the apparently contradictory observations of the two next-generation sequencing studies. Knowing that T-cell repertoires are already dominated by many oligoclonal T-cell clones in blood of melanoma patients before immunotherapy (Figure 1), higher degree of richness and evenness of TCR diversity before treatment (19) may correspond to the TCR repertoires characterized by a large number of T-cell clones. Together with previously observed higher numbers of maintained T-cell clones during CTLA4 inhibition (17), our data on restricted TCR repertoires support the view that pre-existing T-cell clones may be relevant for anti-tumor responses under CTLA4 blockade in melanoma patients, with a possibly greater relevance of CD4 T cells. Together, many different circulating clones, but not a few strongly expanded T-cell clones may favor the clinical responses to CTLA4 inhibition in melanoma patients (19).

When analyzing the TCR data, different methodological aspects have to be considered. Simple differentiation between CD4^+^ and CD8^+^ T cells as done here may already improve resolution for analyzing clinical-relevant immune responses. Careful optimization by minimum pre-amplification is essential to avoid PCR errors for both immunoscope and high-throughput sequencing methods (14, 37, 38). The immunoscope analysis can directly visualize the diversity of clonal T-cell responses for each V-gene family. mRNA-based identification may avoid biases that may occur from the analysis of longer sequences in genomic samples (14). Our data may therefore provide a rationale for evolving analysis strategies to develop blood-based approaches for understanding clinically relevant T-cell responses in cancer patients.

The greater extend of CD4^+^ TCR repertoires restrictions with higher numbers of both restricted Vβ-gene families and prominent peaks reveal broader clonal CD4^+^ T-cell responses in melanoma patients. It may be speculated that the more diversified clonal CD4^+^ T-cell responses in our TCR analyses reflect the diversification of active antigen/neoantigen epitopes, i.e., tumor antigenicity that had actually stimulated T-cell responses in context of each patient's HLA haplotypes. Each (neo-)antigenic peptide may selectively activate oligoclonal T cell populations with the same antigen specificity, which tend to share common features in rearranged Vβ-gene family and CDR3 size (24, 38). T-cell responses against each antigenic epitope may result in select peaks with a dominant length in the spectratypy of a particular Vβ-gene family (24, 39, 40). Higher mutational loads may enhance the antigenicity of tumors so that the patients may gain favorable clinical responses to blockade of CTLA4 or PD-1 (12, 13). Then, it may be supposed that an active T-cell epitope for CD4^+^ or CD8^+^ T cells possibly promotes substantial T-cell expansion which shows in prominent peaks of CD4^+^ or CD8^+^ CDR3 spectratypes. (Neo-)antigenic peptides can stimulate T-cell responses when generated as T-cell epitopes from full-length parent proteins (12, 41, 42). Although several *in silico* prediction tools are available, antigen processing, presentation, and strength of antigenicity for each antigenic epitope are all difficult to predict and need validation experiments for each HLA-class I or class II molecule (12, 13, 41). Assuming that diversity of clonal T-cell responses reflect the breadth of tumor-specific T-cell responses, TCR analyses of blood T cells may provide a non-individualized approach to examine how broadly tumors evoke T-cell responses in cancer patients. This approach may help in predicting anti-tumor responses from checkpoint inhibition.

Cancer patients who experienced severe IrAEs under CTLA4 blockade developed higher numbers of newly expanded T-cell clones (18, 22, 23). The enhanced TCR repertoire restrictions observed in patients with IrAEs after CTLA4 inhibition in our cohort confirm former findings using different methodological approaches (18, 22, 23). Furthermore, Subundhi et al. reported the newly expanded clones correlated with grade 2–3 IrAEs were in CD8^+^ T-cell, but not CD4^+^ T-cell clones in 16 analyzed patients (22). We observed that repertoire changes in CD4^+^ as well as CD8^+^ blood T cells were significantly associated with IrAEs. Considering the limited sample sizes in both studies, it may be too early to attribute IrAEs to the clonality of a select T-cell subset. The degree of clonal expansions may further correlate with the type of IrAEs: our two patients with severe colitis developed stronger repertoire restrictions in CD4^+^ T cells, suggesting that colitis and other IrAEs may differentially involve CD4^+^ and CD8^+^ T cells, though further studies are required.

Collectively, our data demonstrated that the endogenous tumor-related T-cell responses may manifest clonal T-cell populations in blood of patients with advanced melanoma even before initiation of immunotherapy. We show that broader clonal T-cell responses especially in CD4^+^ T cells before immunotherapy, as revealed by more diversified T-cell clones particularly of CD4^+^ T cells, may favor longer survival of melanoma patients with blockade of CTLA4 and that similar conditions may apply to long survivals under PD-1 inhibition as well. Knowing that higher mutational burdens increase tumor antigenicity and likelihood to respond to checkpoint inhibition in melanoma patients (12, 13), we propose that T-cell clones in melanoma patients may reflect the actual tumor antigenicity evoking T-cell expansions in the context of each patient's HLA alleles. As such, analyzing T-cell clones in the blood may enable non-personalized prediction of clinical responses in immune checkpoint inhibition, which is particularly important for this non-individualized treatment. This study shows how extensively T cells can experience clonal expansions after continuous antigenic exposures in the natural clinical course of tumor progression.

## Data Availability

The datasets generated for this study are available on request to the corresponding author.

## Ethics Statement

This study was carried out in accordance with the recommendations of by the Ethics Committee of the Ludwig-Maximilian-University, Munich with written informed consent from all subjects. All subjects gave written informed consent in accordance with the Declaration of Helsinki. The protocol was approved by the Ethics Committee of the Ludwig-Maximilian-University, Munich.

## Author Contributions

Study was conceived and designed by AA and JP. AA, SV, AG, and MB: performed experiments. AA, AG, MH, CB, and JP: collected and analyzed the data. Clinical samples were collected by JT. JT and CB contributed to a draft. AA and JP wrote the manuscript. All authors read and approved the manuscript.

### Conflict of Interest Statement

JT receives speaker's honoraria from BMS, MSD, Novartis, Roche, Almiral, travel support from BMS and consultant honoraria from BMS. CB has served as consultant, investigator, speaker or advisory board member for Amgen, AstraZeneca, BMS, Incyte, Merck, MSD, Novartis, Pierre Fabre, Regeneron, Roche, and Sanofi/Aventis. The remaining authors declare that the research was conducted in the absence of any commercial or financial relationships that could be construed as a potential conflict of interest.

## Abbreviations

CTLA4: cytotoxic T-lymphocyte-associated protein 4
IrAEs: immune-related adverse events
PBL: peripheral blood lymphocytes
PD-1: programmed cell death 1
TCR: T-cell receptor
TILs: tumor-infiltrating lymphocytes.
