# Supplementary material for: Clonality of CD4+ Blood T Cells Predicts Longer Survival With CTLA4 or PD-1 Checkpoint Inhibition in Advanced Melanoma
Source: Front Immunol. 2019 Jun 18;10:1336. doi: 10.3389/fimmu.2019.01336 (PMC6591437; doi:10.3389/fimmu.2019.01336)
Supplement: Supplementary file 1 [file Image_1.pdf]

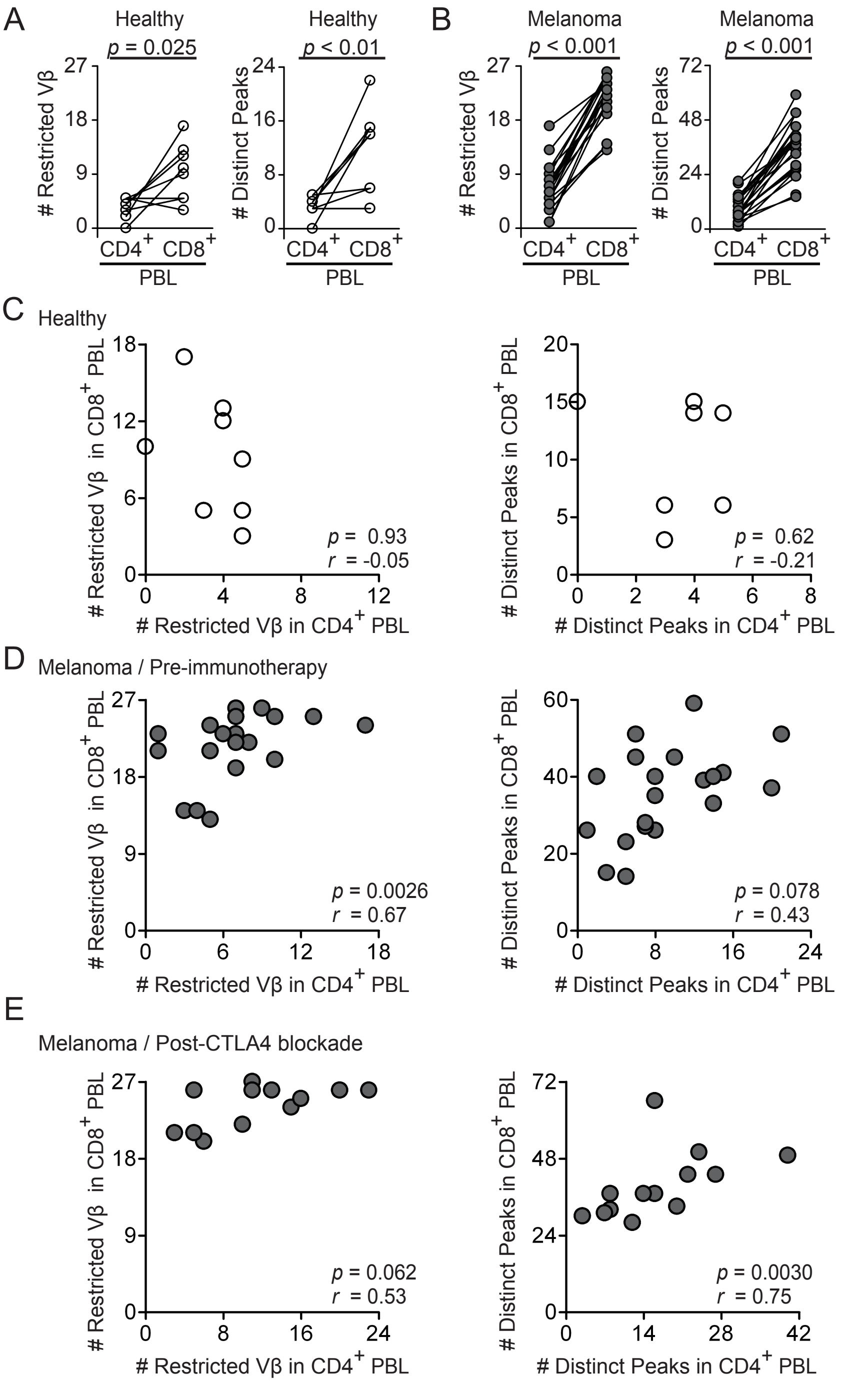

Supplementary FIGURE 1│ Repertoires of CD8+ blood T cells in melanoma patients were more restricted than those of CD4+ T cells.

(**A, B**) Numbers of restricted Vβ-gene families or distinct peaks in pre-immuotherapy spectratypes are compared between CD4+ and CD8+ blood T cells in healthy controls (*A*, $n = 8$) and patients with advanced melanoma (*B*, $n = 20$) by Mann-Whitney *U*-test. Data in **Figures 1 C,D** are re-analyzed. (**C, D**) Intraindividual correlation between restricted Vβ-gene families or distinct peaks of CD4+ and CD8+ blood T cells are analyzed by Pearson-rank correlation test in healthy controls (*C*, $n = 8$) and patients with advanced melanoma (*D,* $n = 20$). Each dot represents one subject. (**E**) Correlations of restricted Vβ-gene families or distinct peaks are analyzed between CD4+ and CD8+ blood T cells after CTLA4 blockade (shown in **Figures 2A,B**) by Pearson-rank correlation test ($n = 13$).

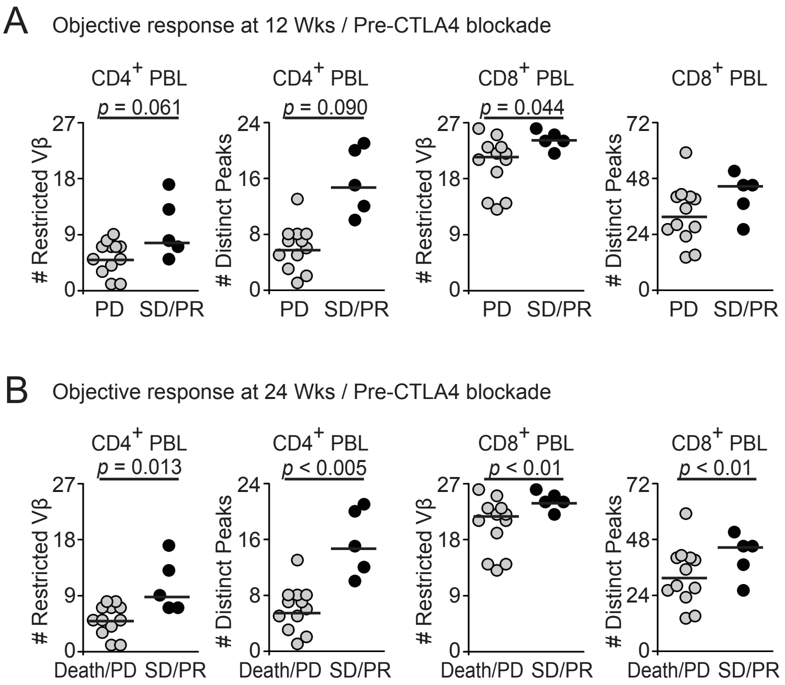

Supplementary FIGURE 2 │ Pretreatment TCR repertoire restrictions were different in patient groups with different objective responses at week 24.

(**A, B**) Numbers of restricted Vβ-gene families or distinct peaks in spectratypes of $CD4^+$ and $CD8^+$ blood T cells are compared between patient groups with different objective responses at week 12 (*A*) or week 24 (*B*) by Mann-Whitney *U*-test. Data in **Figures 1 C,D** are re-analyzed according to objective responses. PD: Progressive disease, SD: Stable disease, PR: Partial response.

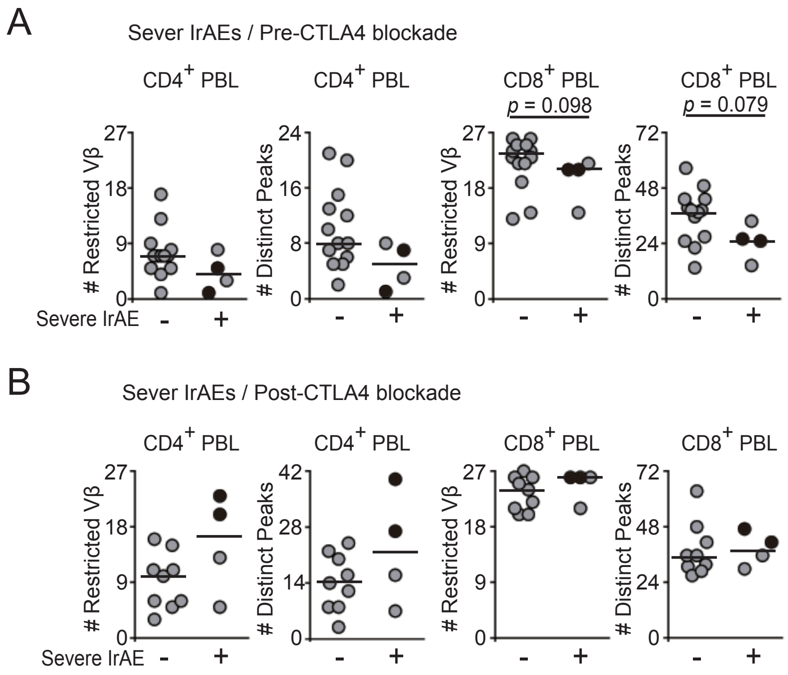

Supplementary FIGURE 3 | Pretreatment CD8$^+$ TCR repertoires tended to be less restricted in patient groups with severe immune-related adverse events (IrAEs).

(**A, B**) Numbers of restricted Vβ-gene families or distinct peaks in spectratypes of CD4$^+$ and CD8$^+$ blood T cells are compared between patient groups without/with severe IrAEs by Mann-Whitney *U*-test. Data in **Figures 1 C,D** are re-analyzed according to IrAEs. Black dot represents patient with severe colitis.
